# Influence of exposure *Heterorhabditis bacteriophora* HP88, (Rhabditida: Heterorhabditidae) on biological and physiological parameters of *Pseudosuccinea columella* (Basommatophora: Lymnaeidae)

**DOI:** 10.1590/S1984-29612023072

**Published:** 2023-11-27

**Authors:** Natânia do Carmo Sperandio, Victor Menezes Tunholi, Ludimila Santos Amaral, Maria Larissa Bitencourt Vidal, Lais Sperandio Cassani, Vinícius Menezes Tunholi-Alves, Melissa Carvalho Machado do Couto-Chambarelli, Jankerle Neves Boeloni, Caio Monteiro, Isabella Vilhena Freire Martins

**Affiliations:** 1 Departamento de Medicina Veterinária, Centro de Ciências Agrárias e Engenharias, Universidade Federal do Espírito Santo – UFES, Alegre, ES, Brasil; 2 Departamento de Biociências e Tecnologia, Instituto de Patologia Tropical e Saúde Pública, Universidade Federal de Goiás – UFG, Goiânia, GO, Brasil; 3 Pós-graduação em Medicina Veterinária, Departamento de Veterinária, Universidade Federal de Viçosa – UFV, Viçosa, MG, Brasil; 4 Pós-graduação em Ciências, Departamento de Parasitologia Animal – DPA, Instituto de Veterinária, Universidade Federal Rural do Rio de Janeiro – UFRRJ, Seropédica, RJ, Brasil

**Keywords:** Biological control, entomopathogenic nematodes (EPNs), fasciolosis, *Lymnaea* sp, Controle biológico, nematóides entomopatogênicos (EPNs), fasciolose, *Lymnaea* sp

## Abstract

Many studies about fasciolosis control have been carried out, whether acting on the adult parasite or in *Pseudosuccinea columella*, compromising the development of the larval stages. The present study aimed to evaluate, under laboratory conditions, the susceptibility of *P. columella* to *Heterorhabditis bacteriophora* HP88, during for 24 and 48 hours of exposure. The snails were evaluated for 21 days for accumulated mortality; number of eggs laid; hatchability rate; biochemical changes; and histopathological analysis. We found that exposure induced a reduction in glucose and glycogen levels, characterizing a negative energy balance, due to the depletion of energy reserves as a result of the direct competition established by the nematode/endosymbiont bacteria complex in such substrates. A mortality rate of 48.25% and 65.52% was observed in the group exposed for 24 h and 48 h, respectively, along with significant impairment of reproductive biology in both exposed groups in relation to the respective controls. The results presented here show that *P. columella* is susceptible to the nematode *H. bacteriophora*, with the potential to be used as an alternative bioagent in the control of this mollusk, especially in areas considered endemic for fascioliasis, in line with the position expressed by the World Health Organization Health.

## Resumo

Muitos estudos têm sido realizados sobre o controle da fasciolose, seja atuando no parasito adulto, seja em *Pseudosuccinea columella*, comprometendo o desenvolvimento das fases larvais. O presente estudo teve como objetivo avaliar, em condições laboratoriais, a suscetibilidade de *P. columela* à *Heterorhabditis bacteriophora* HP88, durante 24 e 48 horas de exposição. Os moluscos foram avaliados por 21 dias para mortalidade acumulada; número de ovos postos; taxa de eclodibilidade; alterações bioquímicas; e análise histopatológica. Constatou-se que a exposição induziu a redução nos níveis de glicose e glicogênio, caracterizando um balanço energético negativo, devido ao esgotamento das reservas energéticas, decorrentes da competição direta estabelecida pelo complexo nematoide/bactéria endossimbionte nesses substratos. Foi observada uma taxa de mortalidade de 48,25% e65,52% no grupo exposto por 24 h 48 h, respectivamente, juntamente com comprometimento significativo da biologia reprodutiva em ambos os grupos expostos em relação aos respectivos controles. Os resultados aqui apresentados, mostram que *P. columela* é suscetível ao nematódeo *H. bacteriophora*, com potencial para ser utilizada como um bioagente alternativo no controle dess molusco, especialmente em áreas consideradas endêmicas para fasciolose, em linha com a posição expressa pela Organização Mundial da Saúde.

## Introduction

Snails play an important role in veterinary and human medicine, since they are intermediate hosts (IH) of various parasites, mainly trematodes with zoonotic potential. The gastropod *Pseudosuccinea columella* (Syn. *Lymnaea columella*) has aquatic habits and mainly colonizes stagnant or slow-moving water in several world regions (Mas-Coma et al., 2009). This species participates in the life cycle, as IH, of the fluke *Fasciola hepatica*, the causal agent of fasciolosis, a disease distributed worldwide, which mainly affects the liver and bile ducts of ruminants (cow and sheep) and humans (Bennema et al., 2014).

Among the control and prevention strategies related to fascioliasis, not only the chemical treatment of definitive hosts, but also the adoption of population control measures for the IH snail to reduce cases of infection in endemic areas (Amaral et al., 2022). For many years, the control of these snails has been carried out using chemical molluscicides (Singh et al., 2010), but Cantanhede et al. (2010) reported that the use of such compounds has raised concerns about toxicity to non-target species due to their low selectivity, in addition to the long residual period.

In this sense, biological control arises, involving the use of entomopathogenic nematodes (EPNs), capable of colonizing and killing target host species especially insects, as well as ticks and gastropod snails (Schurkman & Dillman, 2021).Added to this is the fact that nematodes belong to the genus *Heterorhabditis* are naturally isolated froms oils (Ciche et al., 2006). *Pseudosuccinea columella* is considered an “amphibian” snail, with lungs, and can be collected even in soils close to water (Mas-Coma et al., 2009). Thus, allow in gits exposure under natural conditions to *Heterorhabditis*.

The exposure of *P. columella* to *Heterorhabditis baujardi* LPP7 was tested by Tunholi et al. (2017) and Vidal et al. (2021), and both observed interferences linked to reproductive biology and physiological changes in the snail. Although it is known that some species of *Heterorhabditis*, such as *Heterorhabditis indica, H. baujardi* and *Heterorhabditis heliothidis*, are infective in gastropods, we found no studies in the literature that have evaluated the susceptibility of *P. columella* to the species *H. bacteriophora*, strain HP88, so this study is groundbreaking.

The present study the susceptibility of *P. columella* to *H. bacteriophora* HP88 and molluscicidal effect, *in vitro*, of IJs infection in *P. columella*, as well as characterize possible physiological, biological and reproductive changes in these snails at different exposure times (24 and 48 hours).

## Materials and Methods

Specimens of *P. columella* were collected from bovine troughs located in the municipality of Alegre (20° 45' 48” South, 41° 32' 2” West), Espírito Santo, Brazil and kept in aquariums at the Laboratory of Parasitology of the Veterinary Hospital do Center for Agricultural Sciences and Engineering of the Federal University of Espírito Santo (CCAE-UFES). The nematodes *H. bacteriophora* HP88 were provided by the Laboratory for Microbial Control of Arthropods of Federal Rural University of Rio de Janeiro (UFRRJ). For multiplication of EPNs, last-instar caterpillars of *Tenebrio molitor* were used, provided by the Center for Scientific and Technological Development in Phytosanitary Management of Pests and Diseases (NUDEMAFI UFES), following the method proposed by Kaya & Stock (1997).

The formation of experimental groups took place as follows: the control and exposed groups were arranged according to two factors: time of exposure (24 or 48 hours); and days after exposure (7, 14 or 21). Experimental exposure to the nematode was carried out individually using 24-well Elisa plates (model K12-024/KASVI). Each well was filled with a snail, distilled water and the exposed groups, plus 150 infective juveniles (IJs = J3) of the nematode, totaling 2 mL. The plates were then incubated in a BOD oven with temperature and humidity maintained at 25-27ºC and 85%, for 24 or 48h, as indicated for each group.

After exposure, 20 snails from each experimental group were transferred to aquariums. The entire experiment was carried out in triplicate, comprising 20 snails for each replicate, in a completely randomized design, using a total of 720 snails. Analyses regarding mortality and posture were performed every 48 hours until the end of the third week of follow-up. For the biochemical and histological analysis, material was collected at the end of each week. The temporal parameter of three weeks of evaluation was based on previous works carried out by Tunholi et al. (2017) and Vidal et al. (2021).

To avoid the influence of population density on the reproductive and physiologic aspects of *P. columella*, snails that died during the experiment, both from the control group and the treated group, were replaced by others kept under the same conditions, thus maintaining the same number of specimens between the groups and the study period.

The analysis of mortality and reproductive biology took place on alternate days until the end of the evaluation, as well as numbers of egg mass, number of eggs and hatching rate were recorded through direct observation as described by Tunholi et al. (2017).

Tissue dissection of the cephalopodal mass (MC), digestive gland (GD), albumen gland (GA) and hemolymph collection (He) from the snails were performed according to the method of Tunholi et al. (2016) and Vidal et al. (2019), at the end of each evaluation week, with 17 snails from each experimental group (control and exposed; 24 and 48 h).

The hemolymph concentration of free glucose was measured by the glucose oxidase method(Doles®). The determination of the content of glycogen stored in the tissues of the (MC) and (DG) was performed according to Pinheiro & Amato (1994), through the adapted 3,5-dinitro-salicylate (DNS) method. The biochemical analysis of the mentioned tissues was carried out in partnership with staff of the Cellular and Molecular Biology Laboratory of the Department of Pharmaceutical Sciences, Faculdade Multivix.

For histological analysis, at the end of each week, three snails from each group were processed as described by Vidal et al. (2019) and stained with hematoxylin-eosin (HE), Masson's trichrome and reactive periodic acid Schiff (PAS). Images were captured using an Opticam Microscopy Technology 055R LOP14003 microscope.

To compare the biochemical and reproductive parameters, the data were first submitted to the Kolmogorov-Smirnov normality test, followed by ANOVA and comparison of means by the Tukey-Kramer test. All statistical calculations were performed with GraphPad Prism 9. The statistical decisions were taken at the significance level α<0.01 (1%). P-values smaller than the significance level will be declared significant for rejecting the tested hypotheses. The results are presented as mean ± standard deviation for biochemical data and as mean ± standard error for reproductive data. The mortality result was obtained from the average of two repetitions, and presented in percentage, through the accumulated mortality rate. The histopathological evaluation was performed from the descriptive analysis of data.

## Results and Discussion

Experimental exposure to *H. bacteriophora* HP88 induced mortality in *P. columella*, being this observed at different exposure times. Among the experimental groups, the one consisting of snails exposed for 48 hours had the highest accumulated mortality rate (65.52% - after three weeks; *P*<0.01) when compared to the control group (18.94%; *P*<0.01), while in the exposure group of 24 hours, the maximum observed mortality was 48.28% (after three weeks; *P*<0.01).

Such findings are in agreement with those reported by Tunholi et al. (2017), who observed that exposure of *P. columella* to *H. baujardi* LPP7 resulted in a mortality rate of 66.66%, a value very close to that obtained at the end of the third week of analysis in the group exposed for 48h (65.52%). In addition, Tunholi et al. (2014) reported 55% mortality in *B. similaris* exposed to *H. indica* LPP1.

As for reproductive infections, about the amount of ovigerous masses in the experimental groups, there was a statistical difference only in the third week of analysis for the group exposed for 48 hours (4.28±0.36; *P*<0.01) in relation to their respective control groups (2.00±0.62; *P*<0.01), demonstrating an increase of 53.27% (Figure S1A supplementary material). The same trend of variation was observed for the number of eggs laid (59.71±7.39 in the exposed group and 28.50±8.60 in the control group) (Figure S1B supplementary material). However, for the hatchability rate, a statistical difference was verified between the group exposed for 24 h (44.65±14.16; *P*<0.01) and the control (77.30±13,48; *P*<0.01) at the end of the third week of analysis; and for the 48 hours group in the second (35.73±10.88;*P*<0.01) and third (42.48±5.50; *P*<0.01) weeks of evaluation, when compared to the controls (65.60±10.20; 62.52±10.10 respectively) (Figure S1C supplementary material).

The analysis of the number of ovigerous masses and of total number of eggs laid by snails revealed there was statistical difference only end of the third week for snails exposed for 48 h, characterizing increased reproductive performance of these snails. This phenomenon was described by Minchella (1985), which he called “fecundity compensation”, a condition in which the parasitized animal increases its reproductive efforts to compensate for future losses that will occur as a result of parasitism.

When analyzing the hatchability rate, it is possible to notice that the eggs of snails that were exposed to EPN did not hatch in the same proportion as the control group, mainly in the third week of analysis for the group exposed to 24 hours and in the last two weeks for the exposed group for 48 hours. This change can be partly explained by the negative energy balance developed by the infected snails, compromising the composition of the peri-vitelline fluid, mainly of galactogen, and therefore, the nutrition of the embryos.

In this sense, Tunholi-Alves et al. (2012) stated that the decrease in reproductive performance observed in *B. glabrata* when parasitized by *A. cantonensis* resulted from the phenomenon of indirect and partial parasitic castration, which is characterized by the reduction in the values of all parameters analyzed, probably as a result of the depletion of the host snail's energy reserves. This was described by Tunholi et al. (2017) for *P. columella* infected by *H. baujardi* LPP7 and by Amaral et al. (2022), studying the relationship *B. glabrata*/*H. bacteriophora* HP88, corroborating with our results.

As for biochemical changes the results of the present study revealed alterations in the oxidative metabolism of *P. columella* during exposure to *H. bacteriophora*. These alterations were observed during the three weeks of analysis, affecting both the glucose contents in the hemolymph and the glycogen stored in the digestive gland and cephalopodal mass.

The concentration of glucose in the hemolymph of snails exposed for 24 h was significantly lower in the first (20.74±0.26 mg/dl; *P*<0.01) and second week (22.09±3.08 mg/dl; *P*<0.01), when compared to their control group (43.97±4.80 and 44.29±4.12, respectively), however, no difference was observed in the third week. This same trend was observed in the group exposed for 48 hours, when the lowest value occurred in the second week (12.95±1.20 mg/dl; *P*<0.01) statistically differing from its control group (48.15±4.27; *P*<0.01) (Figure S2A supplementary material).

Regarding the glycogen reserves found in the digestive gland of *P. columella* exposed to EPNs, a significant decrease was observed for both groups:24 hours (one week 15.50±1.26; two weeks 23.19±3.15; three weeks 25.45±2.55 mg/dl; *P*<0.01); and 48 hours (one week 15.95±1.25; two weeks 20.10±2.95; three weeks 22.87±3.05 mg/dl; *P*<0.01) in relation to unexposed snails (24 hours – one week 39.98±1.58; two weeks 41.39±1.47; three weeks 41.37±1.63; 48 hours – one week 40.35±1.95; two weeks 39.40±2.50; three weeks 40.55±1.55 mg/dl; *P*<0.01) (Figure S2B supplementary material). However, when analyzing the impact of exposure on the concentrations of this reserve on the cephalopodal mass, only the 48h group showed a significant difference with the control group, with the greatest decrease verified after the third week of exposure (3.84±0.55 mg/dl; *P*<0.01) compared to their control group (22.62±0.37; *P*<0.01) (Figure S2C supplementary material).

These results show there was intense energy demand, possibly resulting from direct competition for glucose. This metabolic condition forced exposed snails to activate the aerobic carbohydrate degradation pathway to obtain energy, such as glycolysis and glycogenolysis, in an attempt to maintain homeostasis. Similar results were verified by Lima et al. (2017) when analyzing the influence of exposure of *A. cantonensis* to *Biomphalaria*. According to them, the depletion of the glycogen observed in the exposed snails occurred in response to the increased withdrawal of nutrients from the hemolymph, mainly free glucose, by the larval stages of the nematode.

In the present study, snails exposed for 24h were able to restore their glucose concentrations to levels similar to those of the control in the third week of analysis, thus suggesting the existence of homeostatic mechanisms involved in the control of glucose, denoting the ability of these hosts to adapt to this physiological stress condition. Such metabolic plasticity was described by Thompson & Lee (1986) in the *B. glabrata*/*Schistosoma mansoni* interface and by Tunholi-Alves et al. (2013) studying the relationship between *B. glabrata* and *A. cantonensis*.

In the group exposed for 48 hours, there was mobilization of glycogen both from the digestive gland and from the cephalopodal mass, however was not sufficient to restore normoglycemia. Thus, it is suggested that the breakdown of glycemic homeostasis observed here may have been due to greater competition for nutrients.

These results are in agreement with those previously published by Amaral et al. (2022), who affirms that such findings result from the direct competition for nutrients between the host snail and the nematode/bacteria complex, since *in vitro* studies have confirmed the ability of *Photorhabdus luminescens* to use glucose as an energy substrate, favoring the hypoglycemic state in the parasitized hosts (Jeffke et al., 2000).

In the histopathological evaluation of the snails in the control group, there were no alterations (Figure S3 supplementary material); In addition, the presence of glycogen was strongly marked by PAS staining, as shown in images A and C. On the other hand, in snails exposed to *H. bacteriophora* HP88, alterations mainly established in the digestive gland were observed (Figure S3D supplementary material), characterized by the weak presence of glycogen, multifocal vacuolation and cell lysis.

Tunholi-Alves et al. (2013) reported that *A. cantonensis* infection also induced important histological changes in the digestive gland of *B. glabrata*. According to the authors, the organ in question has a remarkable ability to synthesize glycogen, proteins and lipids, providing in abundance essential nutrients for the evolution of the larval stages of the nematode, representing the main site of parasitic development. Information provided by Faro et al. (2013) corroborates the data observed in the present study.

In ovotestis, a relevant reduction in the number of reproductive cells was observed, mainly of oogonia and accumulation of amorphous material (Figure S3F supplementary material), while in the albumen gland the atrophy of the glandular tissue was observed (Figure S3H supplementary material). Pan (1965) stated that these changes directly affect the reproductive system, leading to a reduction of reproductive capacity. The results are in agreement with the findings described by Faro et al. (2013) and reveal the appearance of light areas due to the decrease in oogonia cell density. The authors reiterated a reduction in the resources involved in reproduction, leading to interruption of oviposition during the patent period of infection.

In the cephalopodal mass (Figure S3B Supplementary material), a weak presence of glycogen, multifocal vacuolation, cell lysis and structural disorganization was observed. Tunholi-Alves et al. (2013) reported that the reduction in the amount of galactogen stored in the albuminous gland of snails results in a low hatching rate, characterizing the phenomenon of reduced fertility. Galactogen is an isomer of glycogen, being the only polysaccharide that integrates part of the perivitelline fluid of gastropods, used as a source of nutrients in the development of embryos before and after hatching. Thus, in situations of negative energy balance, during parasitism, there is a breakdown of glycemic homeostasis in these hosts, compromising galactogenesis in infected snails. This directly impairs the reproductive performance of these hosts, partly explaining the reproductive changes observed in *P. columella* exposed to *H. bacteriophora.*

In the present study, consolidation of typical inflammatory granulomas was not observed in *P. columella* exposed to *H. bacteriophora*. Our results are in agreement with those previously described by Amaral et al. (2022), studying the relationship between *B. glabrata* and *H. bacteriophora* HP88. The absence of inflammatory reactions was also demonstrated in insects exposed to IJs from EPNs (Ribeiro et al., 2003). According with these authors, the absence of hemocyte recruitment and formation of granulomas results from the degradation of granulocytes by cytotoxins produced by the metabolism of bacteria. However, the data obtained here do not allow us to elucidate which mechanism occurred in the nematode/bacteria complex, justifying the need for further research.

## Conclusions

This is first *in vitro* report of the susceptibility of *Pseudosuccinea columella* to infection by *Heterorhabditis bacteriophora* HP88. The exposure caused considerable physiological disturbances, thus compromising the snail’s homeostatic parameters, reproductive and tissue indices, as well as causing significant mortality. Thus, the histological, physiological and reproductive data suggest that the *P. columella/H. bacteriophora* relationshipis experimentally functional and a potential alternative tool for the biological control of this snail. In this respect, the present study confirms the in vitro pathogenicity of EPNs to snails, describing another species susceptible to infection.

## Supplementary Material

Supplementary material accompanies this paper.

Figure S1. Relation between the number of ovigerous masses (A); number of eggs (B) and hatching rate (C) of *Pseudosuccinea columella* experimentally exposed (24 and 48 h) to *Heterorhabditis bacteriophora* HP88 during three weeks. (a, b) indicates that the means differ significantly from each other at p<0.01.

Figure S2. Comparation between glucose concentrations in the hemolymph (A) and glycogen in the digestive gland (B) and cephalopodal mass (C) of *Pseudosuccinea columella* experimentally exposed (24 and 48 h) to *Heterorhabditis bacteriophora* HP88 during three weeks. Bars followed by the same letters do not vary between each other using the Tukey test at 1% probability.

Figure S3. Photomicrograph of *Pseudosuccinea columella* in HE (E, F, G and H) and PAS (A, B, C and D) staining, showing the control group (A, C, E and G) and exposed group (B, D, F and H). It is observed (A) cephalopedic mass and (C) digestive gland: with high concentration of glycogen (), contrasting the same sites with lower concentration of glycogen (), (B and D respectively) with (B) disorganization of muscle fibers, vacuolization and cell lysis; (D) cysts (  ). (E and F) ovotestis: (E) with many spermatogonia ( ) and oogonia ( ); (F) few germ cells, mainly oogonia; apoptotic oocytes ( ) and accumulation of intracellular amorphous material. (G and H) albumen gland: (G) preserved; (H) atrophy of glandular tissue (*).

This material is available as part of the online article from https://doi.org/10.1590/S1984-29612023072
