# Supplementary material for: Influence of exposure Heterorhabditis bacteriophora HP88, (Rhabditida: Heterorhabditidae) on biological and physiological parameters of Pseudosuccinea columella (Basommatophora: Lymnaeidae)
Source: Rev Bras Parasitol Vet. 2023 Nov 27;32(4):e007023. doi: 10.1590/S1984-29612023072 (PMC10704864; doi:10.1590/S1984-29612023072)
Supplement: Figure S1. [file rbpv-32-4-e007023-Suppl.pdf]

Supplementary Material

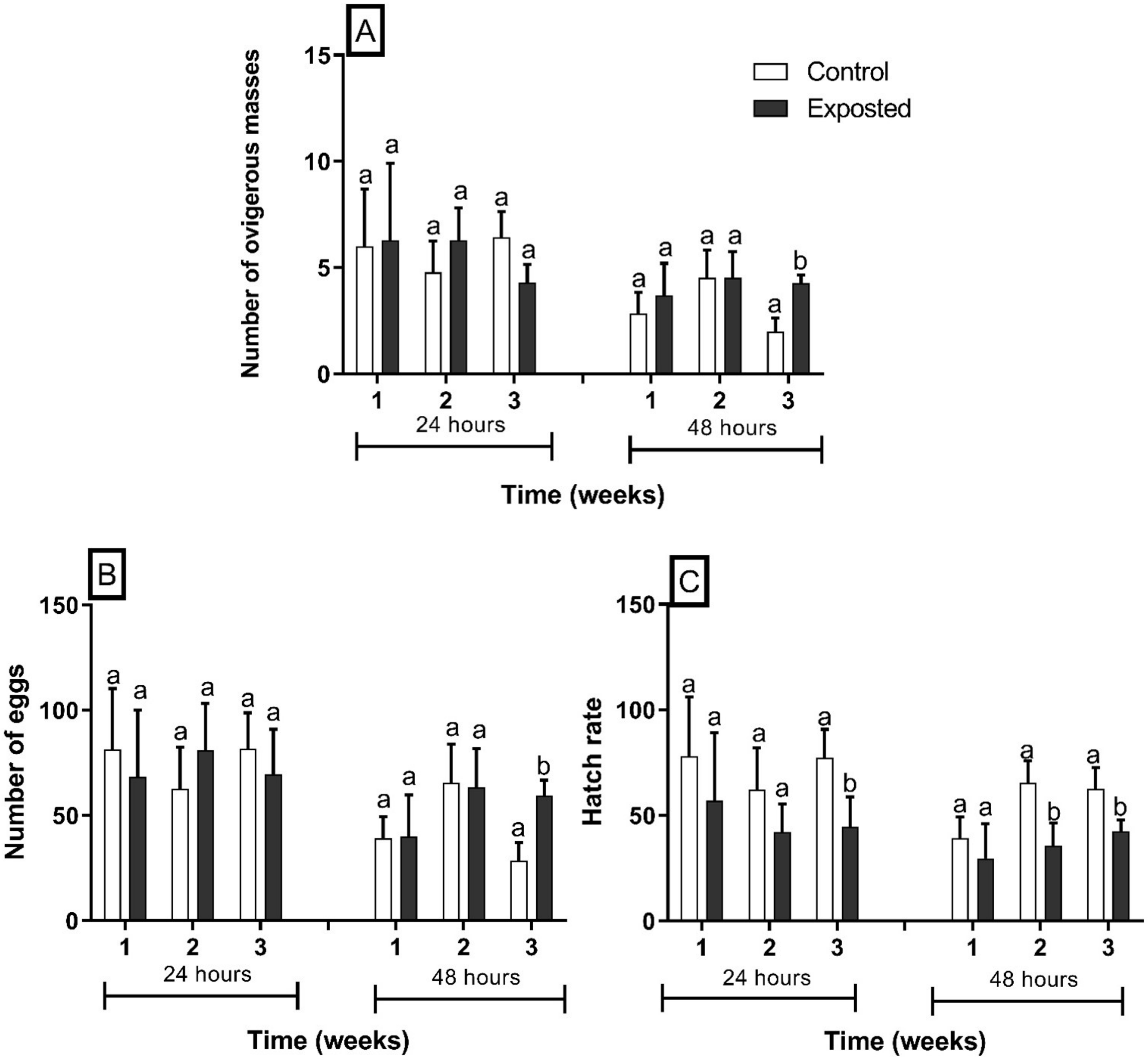

Figure S1 - Relation between the number of ovigerous masses (A); number of eggs (B) and hatching rate (C) of *Pseudosuccinea columella* experimentally exposed (24 and 48 h) to *Heterorhabditis bacteriophora* HP88 during three weeks. (a, b) indicates that the means differ significantly from each other at $p<0.01$.

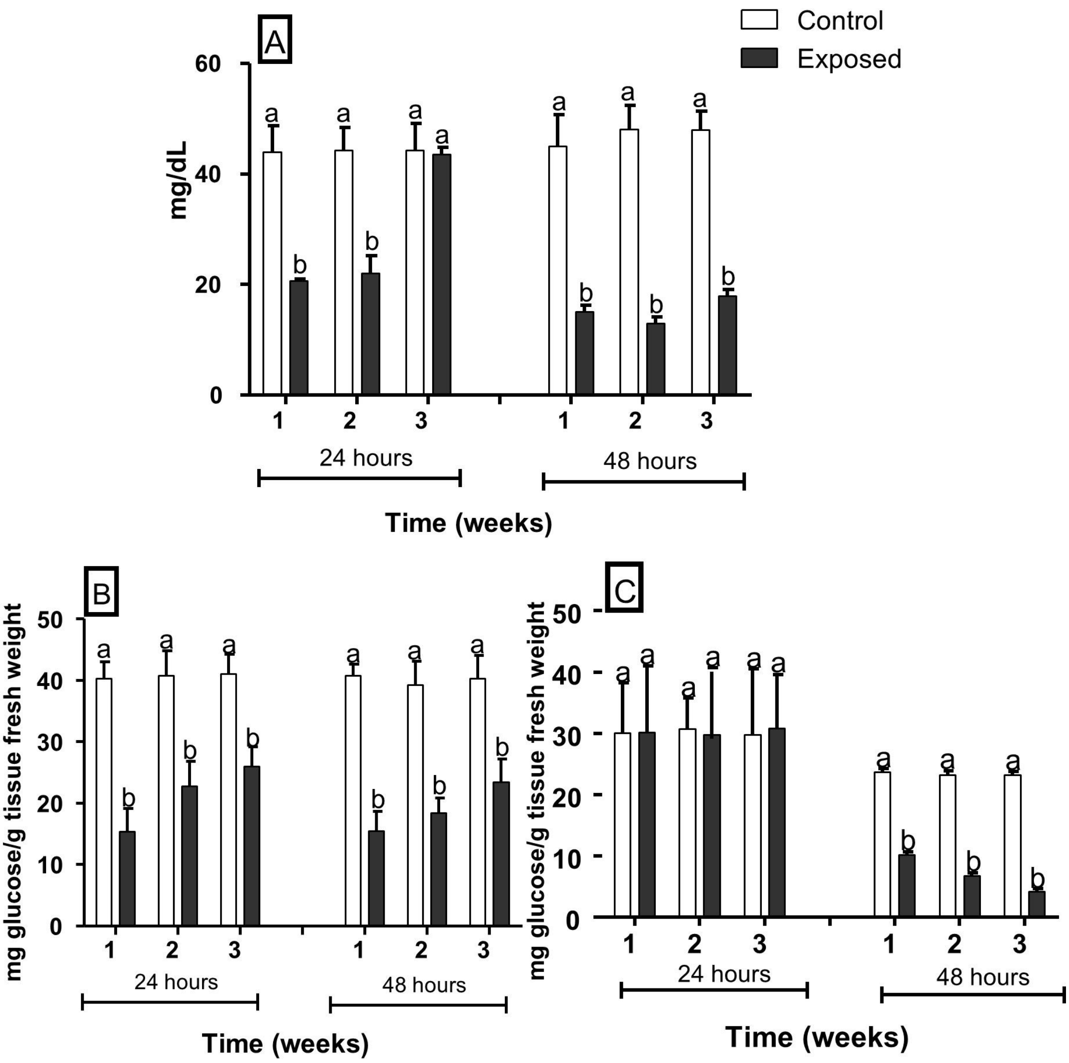

Figure S2 - Comparation between glucose concentrations in the hemolymph (A) and glycogen in the digestive gland (B) and cephalopodal mass (C) of *Pseudosuccinea columella* experimentally exposed (24 and 48 h) to *Heterorhabditis bacteriophora* HP88 during three weeks. Bars followed by the same letters do not vary between each other using the Tukey test at 1% probability

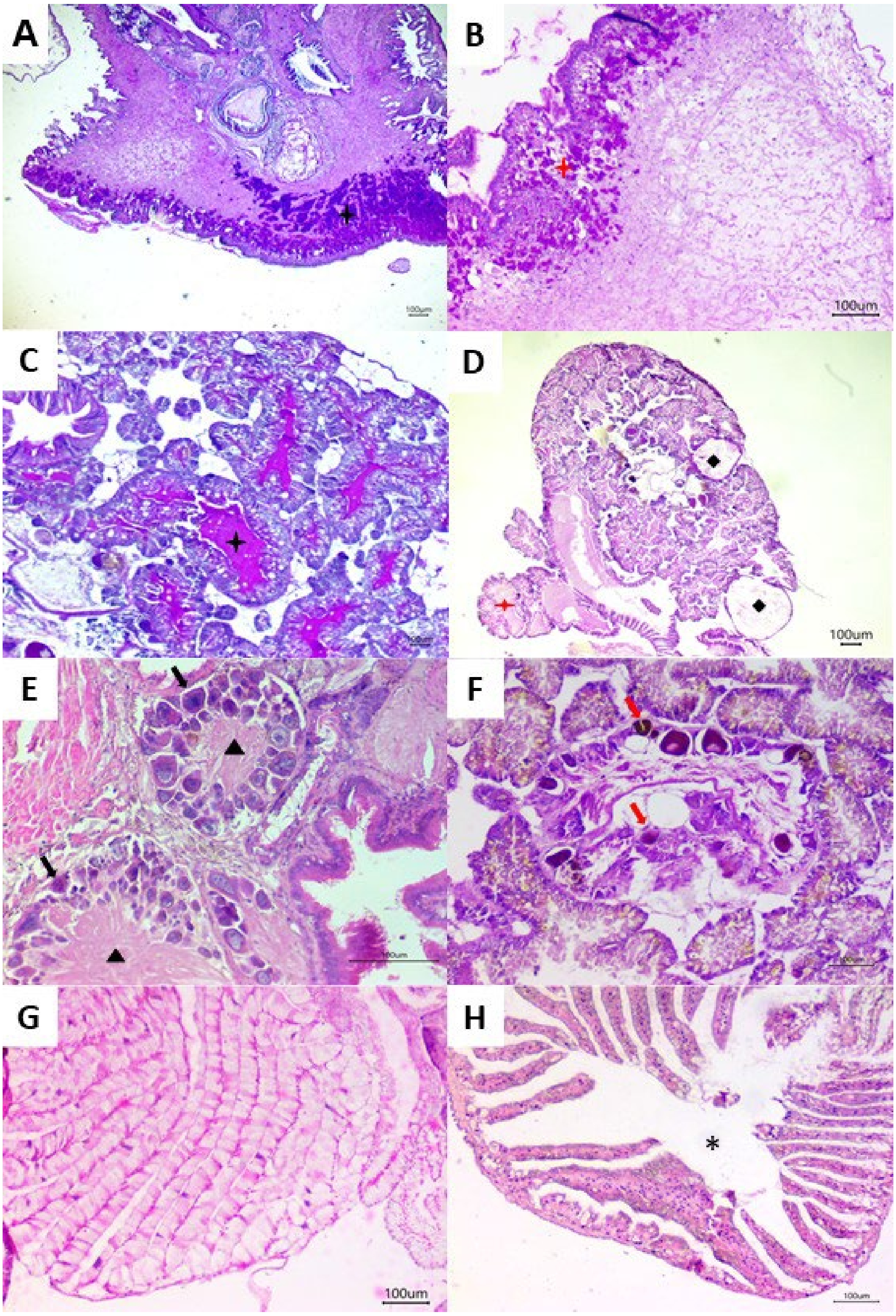

Figure S3 - Photomicrograph of *Pseudosuccinea columella* in HE (E, F, G and H) and PAS (A, B, C and D) staining, showing the control group (A, C, E and G) and exposed group (B, D, F and H). It is observed (A) cephalopedic mass and (C) digestive gland: with high concentration of glycogen (✚), contrasting the same sites with lower concentration of glycogen (✚), (B and D respectively) with (B) disorganization of muscle fibers, vacuolization and cell lysis; (D) cysts (♦). (E and F) ovotestis: (E) with many spermatogonia (▲) and oogonia (➡); (F) few germ cells, mainly oogonia; apoptotic oocytes (➡) and accumulation of intracellular amorphous material. (G and H) albumen gland: (G) preserved; (H) atrophy of glandular tissue (*).
